# The InterVision Framework: An Enhanced Fine-Tuning Deep Learning Strategy for Auto-Segmentation in Head and Neck

**DOI:** 10.3390/jpm14090979

**Published:** 2024-09-15

**Authors:** Byongsu Choi, Chris J. Beltran, Sang Kyun Yoo, Na Hye Kwon, Jin Sung Kim, Justin Chunjoo Park

**Affiliations:** 1 Department of Radiation Oncology, Mayo Clinic, Jacksonville, FL 32224, USA; choi.byongsu@mayo.edu (B.C.); beltran.chris@mayo.edu (C.J.B.); park.chunjoo@mayo.edu (J.C.P.); 2 Yonsei Cancer Center, Department of Radiation Oncology, Yonsei Heavy Ion Therapy Research Institute, Yonsei University College of Medicine, Seoul 03722, Republic of Korea; skyunyoo@yuhs.ac (S.K.Y.); kknh1216@yuhs.ac (N.H.K.); 3 Medical Physics and Biomedical Engineering Lab (MPBEL), Yonsei University College of Medicine, Seoul 03722, Republic of Korea; 4 OncoSoft Inc., Seoul 03776, Republic of Korea

**Keywords:** Swin-Unet, transformer, deep learning, ART, auto-segmentation, head and neck, deform vector

## Abstract

**Simple Summary:**

The InterVision framework employs advanced deep learning techniques to interpolate or create intermediate images between existing ones using deformable vectors, thereby capturing specific patient characteristics, such as unique anatomical features and variations in organ shape, size, and position. These characteristics are vital for personalizing treatment plans in radiotherapy, as they allow for the use of pre-planning information, which is available before the treatment begins, ensuring a tailored and precise approach to each patient’s care. The training process involves two steps: first, generating a general model using a comprehensive dataset, and second, fine-tuning this general model with additional data produced by the InterVision framework. By incorporating the dataset generated through the InterVision framework, we were able to create a more personalized model, surpassing the level of customization achieved by previous fine-tuning approaches. The performance of these models is evaluated using the volumetric dice similarity coefficient (VDSC) and the Hausdorff distance 95% (HD95%) across 18 anatomical structures in 20 test patients. A total of 18 anatomical structures were selected based on prior treatments that involved the most organs, and 20 test patients were chosen according to the availability that has a re-planning CT and manual contours within the total dataset. This framework is especially valuable for accurately predicting complex organs and targets that present significant challenges for traditional deep learning algorithms, particularly due to the intricate contours and the variability in organ shapes across different patients.

**Abstract:**

Adaptive radiotherapy (ART) workflows are increasingly adopted to achieve dose escalation and tissue sparing under dynamic anatomical conditions. However, recontouring and time constraints hinder the implementation of real-time ART workflows. Various auto-segmentation methods, including deformable image registration, atlas-based segmentation, and deep learning-based segmentation (DLS), have been developed to address these challenges. Despite the potential of DLS methods, clinical implementation remains difficult due to the need for large, high-quality datasets to ensure model generalizability. This study introduces an InterVision framework for segmentation. The InterVision framework can interpolate or create intermediate visuals between existing images to generate specific patient characteristics. The InterVision model is trained in two steps: (1) generating a general model using the dataset, and (2) tuning the general model using the dataset generated from the InterVision framework. The InterVision framework generates intermediate images between existing patient image slides using deformable vectors, effectively capturing unique patient characteristics. By creating a more comprehensive dataset that reflects these individual characteristics, the InterVision model demonstrates the ability to produce more accurate contours compared to general models. Models are evaluated using the volumetric dice similarity coefficient (VDSC) and the Hausdorff distance 95% (HD95%) for 18 structures in 20 test patients. As a result, the Dice score was 0.81 ± 0.05 for the general model, 0.82 ± 0.04 for the general fine-tuning model, and 0.85 ± 0.03 for the InterVision model. The Hausdorff distance was 3.06 ± 1.13 for the general model, 2.81 ± 0.77 for the general fine-tuning model, and 2.52 ± 0.50 for the InterVision model. The InterVision model showed the best performance compared to the general model. The InterVision framework presents a versatile approach adaptable to various tasks where prior information is accessible, such as in ART settings. This capability is particularly valuable for accurately predicting complex organs and targets that pose challenges for traditional deep learning algorithms.

## 1. Introduction

Adaptive radiation therapy (ART) has significantly advanced over recent decades. Its key benefit is adjusting treatment plans based on systematic feedback from ongoing measurements [1,2,3,4,5]. This approach enhances radiation therapy by monitoring treatment variations and proactively optimizing treatment protocols as therapy progresses. Online ART further refines this process by adjusting the patient’s treatment plan immediately before delivery, accounting for transient and random changes observed during a single treatment fraction [6,7,8,9]. However, bringing the benefits of online ART faces a significant bottleneck in the recontouring steps. This progress is time-consuming and is a limitation of daily clinic routines.

Conventional techniques, such as atlas-based segmentation and deformable image registration, have been used in the auto-segmentation process to improve the effectiveness and accuracy of the segmentation results [10,11,12,13]. However, these techniques were also not suited to solving the limitations due to the anatomical variations observed during the adaptive treatment. In response, deep convolutional neural networks (DCNNs) have shown the potential to succeed in the segmentation tasks [14,15,16,17,18,19]. Numerous investigations have harnessed the power of CNNs to perform segmentation tasks on various organs and substructures in the context of radiotherapy across different disease sites and imaging modalities. However, given the intrinsic variability in the size of organs at risk (OARs), especially in cases where specific OARs occupy only a few image slices, it is imperative to evaluate the effectiveness of deep neural network models for OAR segmentation within the head and neck (HN) region [20,21].

As CNNs have become a state-of-the-art segmentation method in radiotherapy, more commercial artificial intelligence (AI) software is being introduced into clinical practice. However, a complete subjective and comprehensive evaluation of their performance is lacking. Due to the significant inconsistency between different gold-standard training datasets, the automatic segmentations provided by different vendors vary widely. Different network architectures also lead to discrepancies in prediction results. This increases segmentation uncertainty, and the impact on clinical practice cannot be neglected.

Given the limitations of available datasets in medical imaging compared to those in computer vision, researchers have increasingly adopted the concept of adaptive radiation therapy (ART). Online ART involves repetitive re-planning at each treatment fraction, leveraging prior knowledge from planning CT scans and corresponding contours, either generated by deep learning models or manually created by physicians. This prior knowledge, which closely approximates the ground truth, provides a valuable resource. Many researchers have developed personalized deep learning segmentation (DLS) models through a dual-phase training strategy: initially training a generalized model on a large dataset and then fine-tuning it with patient-specific data [22,23,24,25]. This approach, known as fine-tuning, enhances the model’s accuracy and applicability to individual patients. The transition from general to fine-tuning training is driven by the performance of the general model, which, while robust, may lack precision when applied to individual patient cases. Identified limitations in the general model’s predictions highlight the need for fine-tuning, ensuring that the model adapts more accurately to the unique anatomical features of each patient.

Using the fine-tuning framework, a lot of papers showed a high performance compared to the previous works, but the limitation was that only one dataset was applied, and this resulted into a less personalized fine-tuning model. To solve this problem, other approaches such as using a multi-fraction dataset that can increase personalized dataset collection has been showed. However, if we use the multi-fraction dataset, we cannot use this process in the real-time clinical application because we do not have a multi-fraction dataset before treatment [26,27,28]. To address this challenge, we introduce the InterVision framework, which overcomes the limitations of previous studies by generating new datasets from existing ones, thereby enhancing both the scope and precision of the data. Unlike conventional approaches that rely solely on pre-existing datasets, the InterVision framework creates new datasets by interpolating between existing image slices, generating new images between the slides.

In this study, we have developed an innovative framework designed to generate more personalized datasets by utilizing the original dataset. This enhanced dataset is created using the deformable vector of each slide of the personalized patient. We believe that with further development, and the ability to generate CT images with thinner slice thickness than currently possible, this technique could significantly enhance the creation of more personalized datasets. The primary aim of this research is to propose a patient-specific data augmentation strategy that surpasses the performance of traditional segmentation models. However, we recognize a limitation in that our focus is specifically on head and neck (HN) region segmentation. Ultimately, this approach will enhance the accuracy and robustness of auto-segmentation models, leading to more reliable and precise outcomes in clinical applications.

## 2. Materials and Methods

### 2.1. Patient Cohorts

This study enrolled 120 patients with head and neck (H&N) cancer who underwent radiotherapy (RT). Patients with a history of surgery in the H&N region were excluded to focus on those treated with radiotherapy. This exclusion ensures that the study results are not influenced by surgical interventions, allowing for a more consistent and reliable analysis. All of the CT are scanned using Aquilion TSX-201A (Toshiba, Tokyo, Japan) or Somatom Sensation Open Syngo CT 2009E (Siemens, Munich, Germany) with a slice thickness of 3 mm. From 120 patients, 100 planning CTs (pCTs) and manual contours (MCs) (patients P001–P100) and 20 pCTs and MCs of P101–P120 were the dataset that had a re-planned CT (rpCT) and re-planned manual contours (rpMCs). The rpCT was generated after 36 days (range of 29~43 days). The manual contours were validated from a single radiation oncologist according to the consensus guidelines [29].

### 2.2. Overview of the Framework

**Figure 1:**
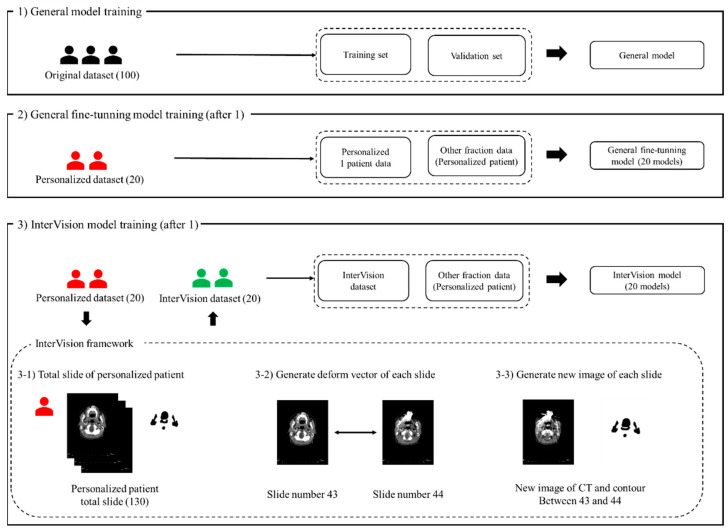
The proposed InterVision framework. (1) illustrates the general model training using the original dataset, the training set and the validation set is divided using the original dataset. (2) illustrates the progress of the general fine-tuning model. The general fine-tuning model is using 1 personalized patient data for the training. For the evaluation, other fraction of the personalized patient data will be used. (3) shows the workflow of the InterVision framework. (3-1), (3-2) and (3-3) show the process of generating InterVision dataset.

Figure 1 illustrates the proposed InterVision framework. The InterVision model consists of two parts: general training and personalized training. As shown in the figure, the initial step involves training a general model using the original dataset. Following this, the personalized model is trained using the dataset generated by the InterVision framework using the personalized dataset. The goal of employing the InterVision framework is to create a gold-standard model tailored for each patient. Given the limitations of the dataset compared to those available in computer vision, this discrepancy makes it challenging to fit them into an adaptive personalized framework. In this study, the InterVision model is aimed to create new images by generating intermediate slices using the deform vector between each original slice. Essentially, for each pair of adjacent slides, a deform vector will be used to produce a new image that represents the transition from one slide to the next. This method allows us to interpolate and generate additional data points, enhancing the overall dataset.

**Figure 2:**
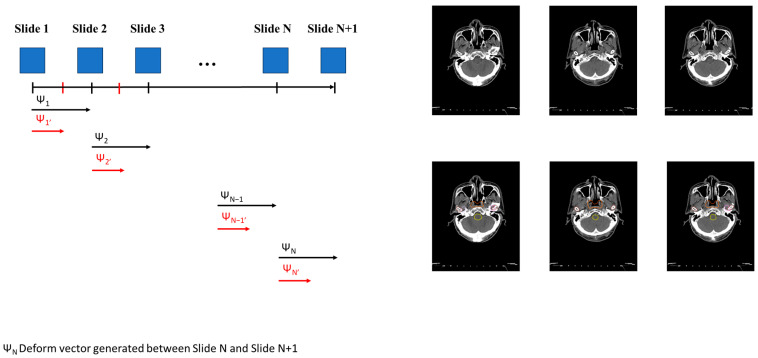
Conceptual representation of generating the InterVision dataset. A deformable vector is created by comparing each slide. Utilizing this deformable vector, we generate intermediate images between each slide. Consequently, we nearly doubled the size of the personalized dataset.

After training the general DL model using the original dataset in Step 1, we generated the fine-tuning model using the personalized model in Step 2. We generated a new image for each slide using the deform vector specific to each slide. This approach allows us to collect a more personalized dataset, making the model better suited for individualized patient care (Figure 1(3-1)). The concept of the InterVision framework is illustrated in Figure 2. Using the dataset generated by the InterVision framework, we have trained an InterVision model to enhance personalization and accuracy in patient-specific applications.

### 2.3. The General Model and the General Fine-Tuning Model

We assume a general dataset consisting of n patients (n = 100), each with a planning CT (pCT). The general model is trained using this dataset of 100 patients, none of whom have a re-planning CT (rpCT). These 100 patients are divided into training and validation sets. For the general fine-tuning model, the personalized dataset is derived from the single planning CT (pCT) and the corresponding manual contour (MC) of the patient of interest. The patient’s re-planning CT (rpCT) and re-planning manual contour (rpMC) are used as the test dataset.

The primary difference between the general model, general fine-tuning model and the InterVision model lies in the data used for training:

- The general model: This model is trained on a standard dataset without any personalized adjustments.
- The general fine-tuning model: This model takes the general model and fine-tunes it using data from a single patient to better adapt to that specific case.
- The InterVision model: This model builds upon the general fine-tuning model by incorporating additional data generated by the InterVision framework.

### 2.4. The InterVision Framework

Before generating the InterVision dataset, we first standardized the image resolution for all patients to 1.0 × 1.0 × 3.0 mm³. Additionally, we adjusted all patient image sizes to 160 × 128 × 130. Subsequently, we generated deform vectors between each slide of the personalized patient images. To create these deform vectors, we developed a Python script based on the reference methodology [30]. Selecting control points was essential for the deformation process. These control points were chosen based on the segmentation results for each organ, comparing the patient slide (N) to another patient slide (N + 1).

(1) $$D_{p}=L_{p}-K_{p},$$

**Figure 3:**
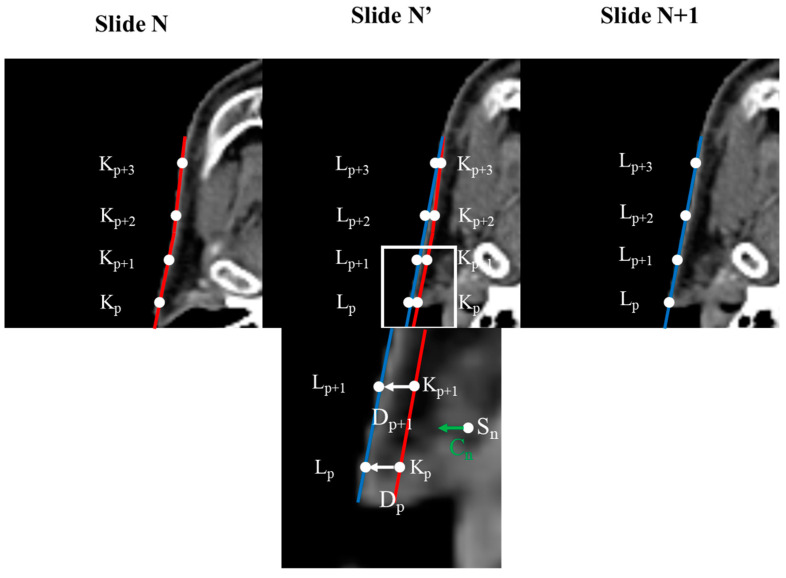
Concept of calculating deformation vectors using control points. Images within the original image are repositioned based on the deformation vectors derived from each control point. The degree of deformation applied to a voxel increases as its proximity to the control point decreases.

$L_{p}$ represents the control point on slide N + 1, $K_{p}$ represents the control point on slide N, $D_{p}$ is the deformation vector from $L_{p}\;$to $K_{p}$, and p is the index of the control point number. Gathering the deformable vector of each slide is illustrated in Figure 3.

Deformable vectors are computed by analyzing the differences between corresponding anatomical structures across adjacent slices. These vectors represent the transformation needed to align the skull structure, capturing the subtle changes in shape and position between the slices. To generate intermediate slices, we interpolate between these deformable vectors, effectively creating a smooth transition that reflects the anatomical continuity. For example, suppose we have two consecutive slices, A and B, the deformable vector would map the control points in slice A to their corresponding positions in slice B by interpolating this vector field. In that case, we can generate an intermediate slice that reflects a gradual shift from A to B, preserving the anatomical details. This process is repeated across the entire patient slides, allowing us to create a more refined and personalized model.

By generating these deformation vector V at the 2D image coordinates, $S_{n}$ was determined through a weighted summation of the deformation vectors corresponding to the control points. This can be mathematically expressed as:

(2) $$V_{n}=\frac{\sum_{p=1}^{m}(G(\left|L_{p}-S_{n}\right|,\sigma_{2})w(\left|L_{p}-S_{n}\right|,\sigma_{1})D_{p}}{\sum_{p=1}^{m}G(\left|L_{p}-S_{n}\right|,\sigma_{2})},$$

(3) $$G\left(x,\sigma \right)=\frac{1}{\sqrt{2}\pi \sigma }\exp\left\{-\frac{x^{2}}{2\sigma^{2}}\right\},$$

(4) $$w\left(x,\sigma \right)=\frac{G\left(x,\sigma \right)}{G\left(0,\sigma \right)},$$

This section may be divided by subheadings. It should provide a concise and precise description of the experimental results, their interpretation, as well as the experimental conclusions that can be drawn.

V represents the deformation vector of 2D image coordinates of the compared image and m is the number of control points. The terms $\sigma_{1}\;$and $\sigma_{2}\;$are the standard deviations, and G denotes the normal distribution. Finally, *w* represents the weight, obtained by normalizing the normal distribution with respect to the central value. Using this approach, we scaled these vector fields by a factor of 0.5 to create intermediate images for each slide. As a result, we doubled up the slides per patient accompanied by paired contours, establishing the foundation for contouring 18 organs.

### 2.5. Model Evaluation

After generating the dataset for training, we proceeded to train and compare three distinct models. The training dataset, consisting of 100 planning CTs (pCTs) and their corresponding manual contours (MCs) from patients P001–P100, was used to develop a generalized auto-contouring model. Following this, the general fine-tuning model and the proposed InterVision model were trained. Basic augmentation techniques were applied across all models to enhance their robustness. For comprehensive validation, a separate set of 20 re-planning CTs (rpCTs) and MCs from patients P101–P120 was employed.

### 2.6. Network Architectures

**Figure 4:**
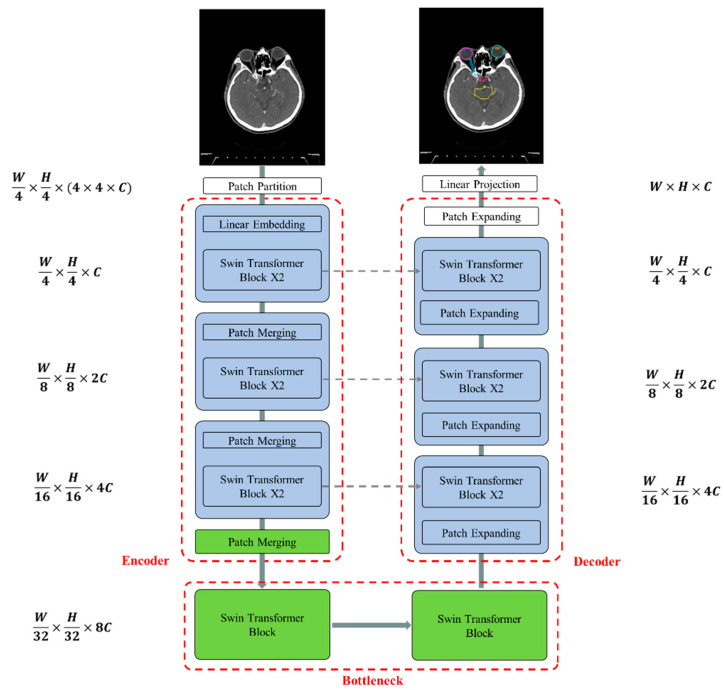
The architecture of Swin-Unet comprises an encoder, bottleneck, decoder, and skip connections. All components—the encoder, bottleneck, and decoder—are constructed using Swin Transformer blocks.

Figure 4 illustrates the overall architecture of the used Swin-Unet [31,32]. Swin-Unet consists of encoder, bottleneck, decoder and skip connections. The basic unit of Swin-Unet is a Swin Transformer block. For the encoder, medical images are divided into non-overlapping patches with a size of 4 × 4 to transform the inputs into sequence embeddings. This partitioning results in a feature dimension of 4 × 4 × 3 = 48 for each patch. A linear embedding layer is then applied to project the feature dimension into an arbitrary dimension, denoted as C. The transformed patch tokens pass through multiple Swin Transformer blocks and patch merging layers to generate hierarchical feature representations. Specifically, the patch merging layer is responsible for downsampling and increasing the dimension, while the Swin Transformer block handles feature representation learning. Inspired by the U-Net architecture, we designed a symmetric transformer-based decoder. The decoder consists of Swin Transformer blocks and patch expanding layers. The extracted context features are fused with multi-scale features from the encoder via skip connections to compensate for the loss of spatial information caused by downsampling. Unlike the patch merging layer, the patch expanding layer is specially designed for upsampling. This layer reshapes feature maps of adjacent dimensions into larger feature maps with 2× upsampling resolution. Finally, the last patch expanding layer performs 4× upsampling to restore the feature maps to the input resolution (W × H). A linear projection layer is then applied to these upsampled features to produce pixel-level segmentation predictions. Each block will be elaborated upon in the following sections.

**Figure 5:**
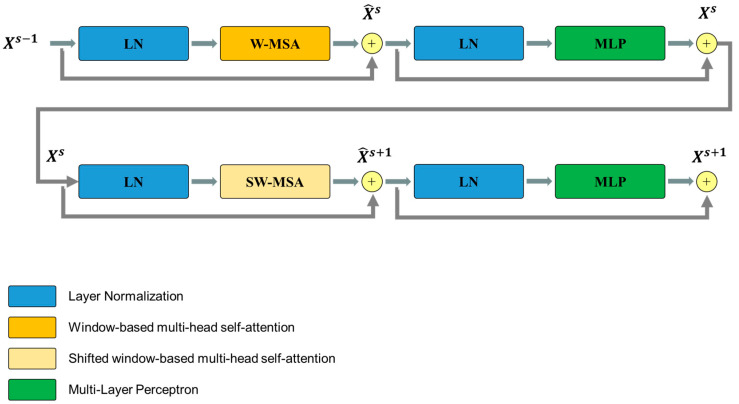
Overview of the Swim Transformer block structure.

The Swin Transformer block is unlike the conventional multi-head self-attention (MSA) module, as the Swin Transformer block is based on shifted windows. As illustrated in Figure 5, two consecutive Swin Transformer blocks are shown. Each Swin Transformer block consists of a LayerNorm (LN) layer, a multi-head self-attention module, a residual connection, and a 2-layer multi-layer perceptron (MLP) with GELU non-linearity. The window-based multi-head self-attention (W-MSA) module and the shifted window-based multi-head self-attention (SW-MSA) module are employed in these successive transformer blocks, respectively. This window partitioning mechanism allows continuous Swin Transformer blocks to be formulated as follows:

(5) $${\hat{X}}^{s}=LN_{1}\left(X^{s-1}\right)+W-MSA(LN_{1}\left(X^{s-1}\right)),$$

(6) $$X^{s}=LN_{2}\left({\hat{X}}^{s}\right)+MLP(LN_{2}\left({\hat{X}}^{s}\right)),$$

(7) $${\hat{X}}^{s+1}=LN_{3}\left(X^{s}\right)+SW-MSA(LN_{3}\left(X^{s}\right)),$$

(8) $$X^{s+1}=LN_{4}\left({\hat{X}}^{s+1}\right)+MLP(LN_{4}\left({\hat{X}}^{s+1}\right)),$$

where ${\hat{X}}^{s}$ and $X^{s}$ represent the outputs of the (S)W-MSA module and the MLP module of the sth block, respectively.

The loss function employed was dual cross-entropy [33]. This dual cross-entropy loss function comprises two components: a cross-entropy term $L_{CE}\;$which is responsible for increasing the probability, and a term $L_{r}$, which is responsible for decreasing the probability. The dual cross-entropy loss function can be expressed as follows:

(9) $$L_{DCE}=L_{CE}+L_{r},$$

(10) $$L_{r}=\frac{1}{M}\sum_{i=1}^{M}({\left(1-y_{i}\right)}^{T}\log\left(\alpha +p_{i})\right),$$

M is the training dataset size, $y_{i}\;$is an ith element of the output vector, and $p_{i}$ is a vector in which jth, element is the probability that sample $x_{i}\;$is assigned to the j th class. $L_{r}\;$aims to improve the model’s generalization by penalizing overconfident wrong predictions, thereby encouraging a more balanced and cautious assignment of probabilities across the different classes.

### 2.7. Evaluation Metrics

We assessed VDSC [34] and the HD95 [35] in three trained models to compare with the MCs. The VDSC is a measure of segmentation volume overlap, comparing the outputs of the trained model segmentation A and the expert segmentation B:

(11) $$VDSC=\frac{2\left|A\cap B\right|}{\left|A\right|+\left|B\right|},$$

The Hausdorff distance is a measure that compares the spatial separation between the trained model segmentation A and the expert segmentation B. Specifically, HD95 represents the largest surface-to-surface separation among 95% of the surface points of the trained model segmentation A and the expert segmentation B. Let a and b denote points in A and B, respectively.

(12) $$HD95\left(A,B\right)={\underset{a\subset A}{\max}\left\{\underset{b\subset B}{\min}\left(dis\left(a,b\right)\right)\right\}}_{95\%},$$

This section may be divided by subheadings. It should provide a concise and precise description of the experimental results, their interpretation, as well as the experimental conclusions that can be drawn. where $dis\left(a,b\right)$ is the Euclidean distance between points a and b.

VDSC is widely used because it provides a measure of overlap between the predicted segmentation and the ground truth, making it a reliable indicator of how well the model captures the shape and size of the target structure. Its value ranges from 0 to 1, with 1 indicating perfect overlap. This makes it an intuitive metric for assessing the accuracy of the segmentation.

HD95%, on the other hand, measures the maximum distance between the boundary points of the predicted segmentation and the ground truth, but it focuses on the 95th percentile of these distances. This metric is particularly useful because it is less sensitive to outliers than the traditional Hausdorff distance, providing a more robust assessment of boundary accuracy. This is critical in medical applications, where small inaccuracies at the boundaries can have significant clinical implications.

We chose these metrics over others, such as precision, recall, or Jaccard index, because VDSC and HD95% provide a comprehensive evaluation of both the overlap and boundary accuracy, which are crucial for ensuring that the segmentation is not only accurate but also clinically viable.

By using these two quantitative evaluations, we were able to validate the performance of three models (the general model, the general fine-tuning model and the InterVision model).

## 3. Results

Table 1 presents the total volumetric dice similarity coefficient (VDSC) of the four models for 18 organs in the head and neck region, including the brainstem, oral cavity, larynx, esophagus, spinal cord, left and right cochlea, mandible, left and right parotid, right and left submandibular gland (SMG), thyroid, left and right optic nerve, optic chiasm, and left and right eye. By training the InterVision model with the inclusion of intermediate slides, we achieved more accurate contour predictions compared to other models. The InterVision model improved VDSC results across all organs compared to the general model and the general fine-tuning model. Additionally, the VDSC standard deviations (SD) were significantly lower than those of the other models, highlighting the consistency and robustness of the InterVision approach.

Notably, the InterVision model showed particular performance gains for small organs, where the general model often struggles. For larger structures such as the mandible and eyes, the differences between the models were less pronounced, but the InterVision approach continued to demonstrate strong performance.

Similarly, Table 1 presents the results for HD95. The InterVision model exhibited the best HD95 performance compared to the other models. The PHL-IDOL approach consistently produced smaller standard deviations, indicating more reliable performance. These results emphasize the superior capability of the InterVision model in accurately identifying and segmenting challenging organs, positioning it as a leading solution for precise organ detection.

**Figure 6:**
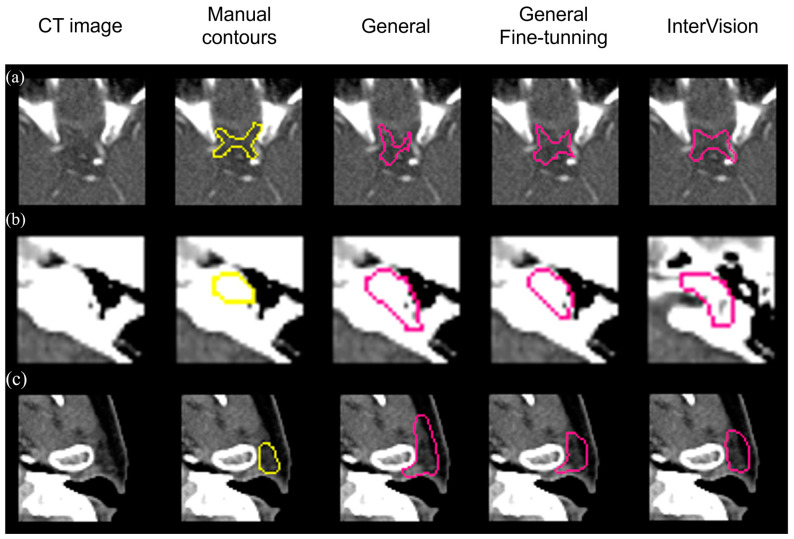
Visual results of the optic chiasm (**a**), L cochlea (**b**) and L parotid (**c**) achieved by the general model, the general fine-tuning model and the InterVision model comparing with the manual contours in yellow.

Figure 6 visually compares segmentation performance. The InterVision model outperforms both the general model and the general fine-tuning model. Remarkably, even in scenarios where the general model struggles the most, the InterVision model excels, particularly in accurately segmenting smaller, harder-to-visualize organs.

## 4. Discussion

By generating accurate contours, we aim to deliver high radiation doses to tumor targets while protecting healthy tissues, a paramount priority in radiation treatment. Consequently, significant research on auto-segmentation using deep learning has been published and remains a hot topic. Despite these advancements, there is a consensus that auto-segmentation models face challenges in clinical applications due to the varied structures of organs at risk (OARs) and the diversity of segmentation algorithms. Additionally, the number of available datasets in the field of medical imaging is significantly lower than in computer vision, which limits progress.

Acknowledging the limitations of available datasets, researchers have turned to adaptive radiation therapy (ART), which involves repetitive re-planning at each treatment fraction by leveraging prior CT scans and contours. This enables the development of personalized deep learning segmentation (DLS) models through a dual-phase strategy: training a generalized model on a large dataset and then fine-tuning with patient-specific data. This method significantly enhances model accuracy and applicability. While fine-tuning has improved performance in many studies, using only one dataset limits personalization. Multi-fraction datasets offer more personalized models but are not feasible for real-time clinical use. To address these issues, we present the InterVision framework, which overcomes these limitations.

The main innovation of the proposed InterVision model develops a framework capable of generating more natural personalized datasets from each slide of the patient. Using each slide of the data, we were able to double up the slides that can overcome the challenge of limited fine-tuning data, resulting in improved segmentation results compared with other models. The InterVision model showed outstanding performance compared to the general model and the general fine-tuning model. The InterVision model demonstrated superior performance with an average VDSC value of 0.85, compared to 0.81 for the general model and 0.82 for the general fine-tuning model. In terms of HD95, the InterVision model achieved the best result at 2.52, while the general model achieved 3.06 and the general fine-tuning model achieved 2.81. Notably, the HD95 result for the InterVision model reflects a significant improvement, with a reduction of 0.54 (nearly a 20% enhancement) over the general model. Additionally, for the VDSC value, most of the contours do not change by more than 6.5% throughout the entire course of radiotherapy, which is comparable to the difference observed between the InterVision model and the general model [36] and comparing with other papers, we believe that the InterVision model shows a significant improvement [25,37].

While the advantages and innovations of the InterVision model are evident, it is important to recognize that the scope of organs examined in this study was limited. To fully validate the framework’s clinical applicability, it is essential to extend our evaluations to encompass target volumes and tumors. Given the model’s exceptional performance in contouring small and difficult-to-visualize organs, there is substantial potential for it to excel in delineating target volumes and tumors [38,39,40]. The results in Table 1 show that the deep learning auto-segmentation models, which typically struggle with accurately predicting the optic chiasm, optic nerve, and cochlea, demonstrated notably improved performance. Building on this success, we are actively developing comprehensive datasets that will include additional critical structures such as the prostate, liver, lung tumors, lymph nodes and clinical target volumes. These structures are chosen for their complexity and clinical significance in radiotherapy. Collaborating with clinical partners, we aim to ensure that our datasets capture a wide range of anatomical variations and treatment scenarios, further validating the robustness and adaptability of the InterVision framework.

Moreover, the realm of online adaptive radiotherapy (ART) presents numerous opportunities where cutting-edge solutions are in high demand. We envisage that our proposed concept can be seamlessly extended to a variety of image generation tasks, including the creation of synthetic CT images from CBCT scans and the enhancement of image resolution from lower-quality inputs. Enhancing these images directly addresses the challenges of real-time treatment adjustments and patient-specific planning, which are essential for better clinical outcomes. We plan to explore these tasks by leveraging the InterVision framework’s capability to generate high-quality, patient-specific data, ensuring more accurate and reliable treatment planning. Our future endeavors will involve evaluating the framework’s adaptability and effectiveness in addressing the critical image generation challenges pivotal to online ART.

Our research signifies a major leap forward in adaptive radiotherapy, providing a more robust and dependable framework for creating patient-specific models. The InterVision approach exemplifies the potential for personalized healthcare within radiotherapy environments. This method’s capability to generate datasets enriched with prior information not only overcomes the limitations of the previous fine-tuning DL model but also paves the way for future exploration for online ART. In essence, the InterVision model emerges as a groundbreaking framework that enhances segmentation accuracy and ensures superior treatment planning and execution in adaptive radiotherapy.

## 5. Conclusions

The InterVision approach utilizes a deep learning (DL) framework to create patient-specific DL networks for common adaptive radiotherapy (ART) applications, demonstrating markedly superior performance over traditional methods. The InterVision concept epitomizes a flexible framework that can be adapted to a multitude of tasks where prior information is available, as is often the case in ART settings. By employing InterVision, we anticipate performance enhancements, with VDSC increasing from 81% to 85% and HD95 results improving from 3.06 mm to 2.52 mm compared to the general model. These advancements lead to more accurate treatment plans, potentially improving patient outcomes by delivering higher radiation doses to tumors while better sparing surrounding healthy tissues. This capability is especially valuable for accurately predicting complex organs and targets that are challenging for DL algorithms.

## Figures and Tables

**Table 1:** Comparison of segmentation performance for 18 organs using the general model, the general fine-tuning model, and the InterVision model using VDSC and 95% Hausdorff distance measures.

		VDSC	SD			VDSC	SD			HD95	SD			HD95	SD
Brainstem	General	**0.88**	**0.01**	L cochlea	General	**0.74**	**0.08**	Brainstem	General	**3.26**	**0.70**	L cochlea	General	**2.39**	**1.01**
	General Fine-tunning	**0.89**	**0.02**		General Fine-tunning	**0.75**	**0.09**		General Fine-tunning	**3.16**	**0.67**		General Fine-tunning	**2.11**	**0.85**
	**InterVision**	**0.90**	**0.02**		**InterVision**	**0.84**	**0.05**		**InterVision**	**2.97**	**0.61**		**InterVision**	**2.01**	**0.81**
Oral cavity	General	**0.89**	**0.02**	R cochlea	General	**0.74**	**0.03**	Oral cavity	General	**4.75**	**1.55**	R cochlea	General	**2.52**	**0.97**
	General Fine-tunning	**0.90**	**0.01**		General Fine-tunning	**0.75**	**0.04**		General Fine-tunning	**4.19**	**0.57**		General Fine-tunning	**2.22**	**0.84**
	**InterVision**	**0.92**	**0.01**		**InterVision**	**0.79**	**0.03**		**InterVision**	**3.78**	**0.43**		**InterVision**	**1.89**	**0.49**
Larynx	General	**0.85**	**0.03**	Mandible	General	**0.94**	**0.01**	Larynx	General	**3.15**	**0.58**	Mandible	General	**1.4**	**0.45**
	General Fine-tunning	**0.85**	**0.03**		General Fine-tunning	**0.95**	**0.01**		General Fine-tunning	**3.12**	**0.55**		General Fine-tunning	**1.29**	**0.39**
	**InterVision**	**0.88**	**0.01**		**InterVision**	**0.95**	**0.01**		**InterVision**	**2.97**	**0.32**		**InterVision**	**1.21**	**0.34**
Esophagus	General	**0.81**	**0.05**	L optic nerve	General	**0.71**	**0.05**	Esophagus	General	**4.6**	**1.51**	L optic nerve	General	**2.94**	**1.47**
	General Fine-tunning	**0.82**	**0.03**		General Fine-tunning	**0.73**	**0.06**		General Fine-tunning	**4.13**	**1.01**		General Fine-tunning	**2.57**	**1.29**
	**InterVision**	**0.85**	**0.03**		**InterVision**	**0.77**	**0.05**		**InterVision**	**3.5**	**0.77**		**InterVision**	**2.21**	**0.53**
Spinal cord	General	**0.81**	**0.04**	R optic nerve	General	**0.70**	**0.07**	Spinal cord	General	**2.38**	**0.85**	R optic nerve	General	**2.82**	**1.84**
	General Fine-tunning	**0.83**	**0.04**		General Fine-tunning	**0.74**	**0.07**		General Fine-tunning	**2.13**	**0.33**		General Fine-tunning	**2.46**	**1.33**
	**InterVision**	**0.86**	**0.03**		**InterVision**	**0.76**	**0.06**		**InterVision**	**2.07**	**0.21**		**InterVision**	**2.08**	**0.61**
L parotid	General	**0.81**	**0.05**	Optic chiasm	General	**0.49**	**0.19**	L parotid	General	**3.58**	**0.45**	Optic chiasm	General	**3.96**	**1.78**
	General Fine-tunning	**0.85**	**0.03**		General Fine-tunning	**0.51**	**0.15**		General Fine-tunning	**3.27**	**0.41**		General Fine-tunning	**3.45**	**1.39**
	**InterVision**	**0.88**	**0.02**		**InterVision**	**0.60**	**0.17**		**InterVision**	**2.8**	**0.38**		**InterVision**	**3.07**	**1.24**
R parotid	General	**0.87**	**0.03**	L eye	General	**0.90**	**0.02**	R parotid	General	**3.74**	**1.13**	L eye	General	**2.17**	**0.47**
	General Fine-tunning	**0.89**	**0.02**		General Fine-tunning	**0.91**	**0.02**		General Fine-tunning	**3.33**	**0.79**		General Fine-tunning	**2.10**	**0.43**
	**InterVision**	**0.90**	**0.01**		**InterVision**	**0.93**	**0.01**		**InterVision**	**2.93**	**0.35**		**InterVision**	**2.00**	**0.39**
R SMG	General	**0.82**	**0.05**	R eye	General	**0.89**	**0.01**	R SMG	General	**3.26**	**1.29**	R eye	General	**2.26**	**0.55**
	General Fine-tunning	**0.84**	**0.02**		General Fine-tunning	**0.90**	**0.02**		General Fine-tunning	**3.12**	**0.78**		General Fine-tunning	**2.16**	**0.47**
	**InterVision**	**0.86**	**0.01**		**InterVision**	**0.93**	**0.01**		**InterVision**	**2.65**	**0.49**		**InterVision**	**2.01**	**0.29**
L SMG	General	**0.83**	**0.03**					L SMG	General	**3.04**	**0.55**				
	General Fine-tunning	**0.86**	**0.04**						General Fine-tunning	**3.13**	**0.81**				
	**InterVision**	**0.87**	**0.02**						**InterVision**	**2.87**	**0.39**				
Thyroid	General	**0.86**	**0.09**					Thyroid	General	**2.91**	**3.11**				
	General Fine-tunning	**0.87**	**0.04**						General Fine-tunning	**2.67**	**0.97**				
	**InterVision**	**0.89**	**0.03**						**InterVision**	**2.33**	**0.42**				

## Data Availability

The datasets generated during the current study will be available from the corresponding author upon reasonable request.
